# Training exercises, match demands, and variability in elite futsal

**DOI:** 10.3389/fspor.2025.1553046

**Published:** 2025-03-27

**Authors:** João Nuno Ribeiro, António Ferraz, Bruno Gonçalves, Carlos Serrano, Diogo Coutinho, Rafael Ballester, Bruno Travassos

**Affiliations:** ^1^ Department of Sport Sciences, Universidade da Beira Interior, Covilhã, Portugal; ^2^ Polytechnic Institute of Guarda, School of Education, Communication and Sports, SPRINT, Sport Physical Activity and Health Research & Innovation Center, Guarda, Portugal; ^3^ Research Center in Sports Sciences, Health Sciences and Human Development, CIDESD, Vila Real, Portugal; ^4^ CIFD, Sports Research, and Training Center, Jean Piaget University of Angola, Luanda, Angola; ^5^ Departamento de Desporto e Saúde, Escola de Saúde e Desenvolvimento Humano, Universidade de Évora, Évora, Portugal; ^6^ Comprehensive Health Research Centre (CHRC), University of Évora, Évora, Portugal; ^7^ Portugal Football School, Portuguese Football Federation, Oeiras, Portugal; ^8^ Universidad Europea de Madrid, Department of Sports Sciences, Faculty of Medicine, Health and Sports, Madrid, Spain; ^9^ Department of Physical Education and Sports Sciences, University of Maia (UMAIA), Maia, Portugal; ^10^ Department of Physical Education and Sport Sciences, Catholic University of Valencia “San Vicente Martir”, Valencia, Spain

**Keywords:** performance, physical demands, techical-tactical training, game analysis, load variance

## Abstract

**Introduction:**

Understanding the physical demands of different training exercises is crucial for optimizing athlete performance. This study aims to compare high-intensity activities across different training exercises and official match-play scenarios. The analysis focuses on performance variations across training exercises and the variability observed within each format.

**Methods:**

An observational study was conducted on a team competing in the 1st Division of the Spanish futsal league over three consecutive seasons. The training and match sessions were organized into mid-court exercises (20 × 20 m), three-quarters court exercises (28 × 20 m), and full-court exercises (40 × 20 m) and match.

**Results:**

Results showed that the full court exercises presented the more demanding results, and the mid court exercises the less demanding results of external load for both acceleration, deceleration and sprints. The comparison between the training exercises and the match revealed distinct physical demands variability across exercise categories. Mid-court training exercises exhibited significantly higher variability compared to all other training formats and match scenarios. In contrast, match conditions demonstrated the lowest variability.

**Discussion:**

Findings highlight the importance of understanding the physical demands of each training exercise to balance intensity, variability, and contextual relevance to tailor training programs that ultimately optimizing athlete preparation for the multifaceted demands of competitive play.

## Introduction

Over the last years, the development of technological systems for monitoring training and competition has significantly expanded our understanding of the physical demands of futsal. These advancements have facilitated a more detailed characterization of match demands, which can be categorized into external (e.g., total distance, high-intensity running, accelerations and decelerations) and internal (e.g., heart rate, perceived exertion) load metrics (1–3). Moreover, these tools have enabled the identification of the most demanding training exercises (4, 5). In general, futsal match play is characterized by high-intensity intermittent efforts, alternating between high-intensity running, braking, and directional changes interspersed with lower-intensity recovery periods (3, 6). Consequently, players must develop power, strength, and agility to optimize their ability to execute these movements effectively (7). Furthermore, the ability to run at high speeds and accelerate/decelerate emerged as the variable most strongly correlated with other external load metrics and the most significant predictor of distinct activity profiles. This means that these variables effectively differentiate players based on their capacity to perform high-intensity activities (1). In line with this, Rico-González, et al. (2) applied Principal Component Analysis (PCA) to analyze positional metrics that discriminate futsal playing profiles. Results revealed that accelerations and decelerations (2, 8–11) were the most critical metrics for pivots and wingers, whereas for defenders, the key metrics included distances covered at medium (6–12 km/h) and high intensity (18–21 km/h), along with the number of landings per minute.

Regarding training exercises, the manipulation of some task constraints, such as the space of play, the number of players, or the numerical relation between teams constrains the physical demands of training exercises with implications for the players' physical load Under this scope, it is crucial to classify different exercises (2) to characterize the regular stimuli applied to futsal players and identify how these are managed to achieve specific goals (12). Different training exercises are expected to produce varying outcomes (2, 13). Examining the dose-response effect enables the analysis of how specific training exercises individually impact players' performance (14). For example, a previous study in youth futsal compared the physical demands of different training exercises structures with those of match play (4). The study observed that larger relative areas per player were associated with higher external and internal loads, while smaller relative areas promoted more collective adaptations with lower physical and physiological impacts. This knowledge can assist coaches in periodizing training loads during the microcycle, aligning tactical objectives with physical preparation. Additionally, a recent study in rink hockey compared different training drill categories with match play and, unexpectedly, observed that training exercises were significantly less demanding than match play (13). This highlights the importance of comparing the demands of training and matches, a topic that, to our knowledge, has not yet been thoroughly explored in futsal literature.

Traditional approaches to managing training loads in team sports often rely on central tendency measures, overlooking the multifactorial nature of team sport performance (15). This performance is characterized by its stochastic and inherently unstable nature, which often results in overlooking both intra-individual and inter-variability among players in each task (16). Nevertheless, it is crucial to account for the variability of the physical demands of players during both training sessions and match play (17). The principle of “one size doesn't fit all” highlights the importance of individualized approaches in creating and supervising training drills—an especially challenging task in team sports. To address these challenges, this study aims to compare high-intensity activities, such as accelerations, decelerations, and high-speed running, across different training exercises and official match-play scenarios. The training drills are categorised based on three field dimensions (mid-court, three-quarters court, and full court) to assess not only the performance variables but also the variability in these high intensity activities. Based on previous research, full-court training exercises are expected to impose greater physical demands than smaller-sided exercises, while mid-court exercises are anticipated to exhibit the highest variability and matches the lowest.

## Materials and methods

### Study design

An observational study was conducted on a team competing in the 1st Division of the Spanish futsal league over three consecutive seasons: 2020–2021, 2021–2022, and 2022–2023. The training and match sessions were organized into different categories, which were classified according to previous literature (2) and outlined with specific criteria (see Table 1). The categories included official matches, mid-court exercises (20 × 20 m), three-quarters court exercises (¾ court, 28 × 20 m), and full-court exercises (40 × 20 m). During the data collection process, technical staff were not provided with any instructions regarding exercise selection or in-match coaching decisions.

**Table 1:** Court dimension and task description for each analyzed category.

Court dimension	Task description
Match (40 × 20 m)	✓ Matches monitored during the official competition.
Mid-court (20 × 20 m)	✓ Game with/without goalkeeper in 2vs2; and 3vs3 formats
	✓ Game with/without goalkeeper in 2vs2; and 3vs3 formats with outfield player joker
	✓ Exercises involving outfield goalkeeper attacking and defensive actions
Three-quarters court (28 × 20 m)	✓ Game with goalkeeper in 2vs2; 3vs3; and 4vs4 formats with/without players joker
Full court (40 × 20 m)	✓ Situations with numerical superiorities/inferiorities with goalkeeper (2 × 1, 3 × 1 and 3 × 2)
	✓ Games training with goalkeeper (4vs4 format)

**Table 2:** Description of external load variables that characterize high intense activities in futsal.

Type	Variable	Unit	Description
Mechanical	High-Intensity Accelerations	ACC (m/min^−1^)	Total distance covered in positive speed changes per minute [3 to 10 m/s^2^]
	High-Intensity Decelerations	DEC (m/min^−1^)	Total distance covered in negative speed changes per minute [−10 to −3 m/s^2^]
Kinematic	Sprint	Sprint (m/min^−1^) (m/min^−1^)	Total distance running above 18 km/h per minute

### Participants

Although the study initially involved 28 professional futsal players, only 14 players (age: 29.07 ± 4.23 years, height: 1.78 ± 0.06 m, weight: 73.28 ± 4.30 kg) were included in the final analysis, as only those with complete data across all training categories and matches were retained. Observations missing data in any category were entirely excluded to avoid potential biases arising from incomplete data. In total, 1.576 observations were recorded, distributed as follows: match (292), mid-court (367), three-quarters (615), and full court (302).

Goalkeepers were excluded from the study. Each player was given a unique identifying code to protect the privacy of their information, and the statistics were collected as part of the club's performance analysis methodology. The club provided written informed consent for the use of this data for research purposes. The study was approved by the Universidade da Beira Interior's Ethics Committee (CE-UBI-Pj-2020-043). Data collections were carried out according to the international ethical standards with humans based on the Declaration of Helsinki (18).

### Procedures

**Figure 1:**
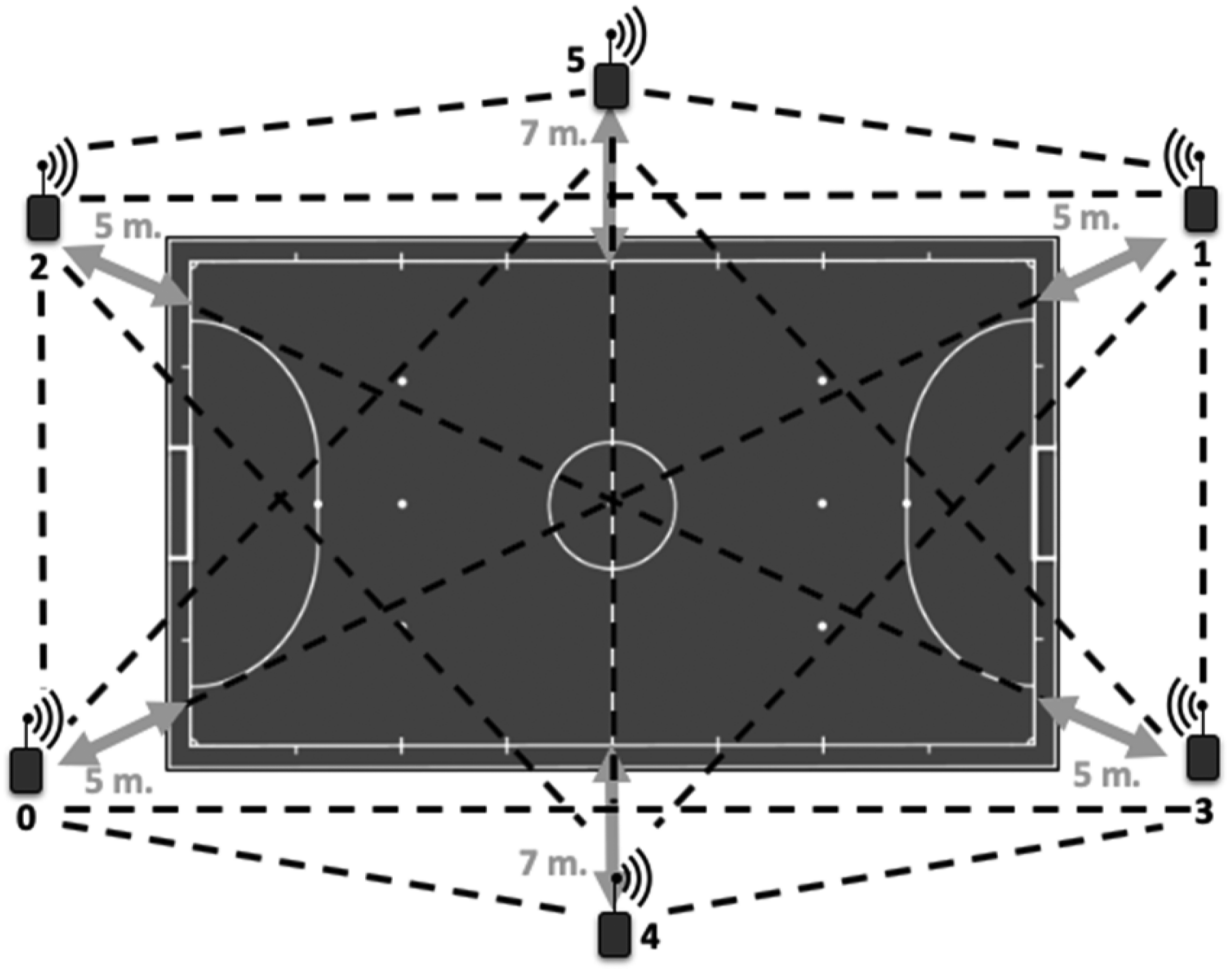
Antenna distribution of the local positioning system and distance reference to the futsal pitch (3).

Data collection of players' activities during training was conducted using the WIMU PRO™ device (Realtrack Systems SL, Almeria, Spain). This device incorporates Inertial Measurement Units (IMUs), Ultra-Wideband (UWB) technology with Location Positioning System (LPS), and a heart rate monitor (Garmin Ltd., Olathe, KS, United States). The Local Positioning System (LPS) was installed on the futsal pitch following the user manual and previous studies (Figure 1). Six UWB antennas were positioned 5 meters from the perimeter line of the field, except for those placed at the midfield line, which were set at 7 meters. This configuration formed a hexagon layout to optimize signal transmission and reception. Once installed, the antennas were activated sequentially, with the master antenna being switched on last. A 5 min autocalibration process then synchronized all antennas to a common clock. Subsequently, the tracking devices were powered on, initiating a 1 min recognition and automatic communication process with the antennas. To establish the court perimeter within the SPRO™ software, a researcher equipped with a tracking device walked along the pitch lines. Finally, each player was fitted with an individual tracking device, secured on the upper back using a specially designed adjustable vest, before the match commenced. Additionally, training sessions were recorded with a video camera (Sony, Tokyo, Japan). All recorded data were then downloaded and processed using the designated software (SPRO™ by Realtrack Systems SL, version 989). The data were synchronized with the video to align active times and participation in the exercises accurately.

### External load variables

The external load data was collected based on the mechanical and cinematic metrics already described as high-intensity actions in futsal (1, see Table 2). Additionally, data were expressed as relative values per minute (m/min), with recordings limited to the time players were actively engaged in the exercise.

### Statistical analysis

After preliminary inspections for the distribution and assumptions, repeated measures ANOVA with Bonferroni *post hoc* analysis was conducted to assess pairwise comparisons between training categories and match performance on the considered variables. Eta Square was calculated to determine the effect size. The thresholds were set at 0.01, 0.06, and 0.14, corresponding to small, medium, and large effects. The statistical analysis was performed using the Statistical package Jamovi (Version 1.8, 2021), and statistical significance was set at *p* < 0.05.

An estimation techniques approach was used to address the limitations of traditional null hypothesis significance testing. Cohen's *d*_unbiased_ (*d*_unb_) was applied along with the 95% confidence interval (CI) to determine the effect size (ES). This unbiased estimate has a sampling distribution whose mean equals the population parameter being estimated. Pairwise differences between the categories were identified using this effect size. The thresholds for effect size statistics were set at 0.2, 0.5, and 0.8, corresponding to small, medium, and large effects.

## Results

Table 3 displays descriptive data of the performance variables and comparisons across match and exercise categories.

**Table 3:** Descriptive statistics (mean ± SD) and repeated measures ANOVA results for performance variables across match and exercise categories.

Performance variables	Match	Mid-court	¾ court	Full court	F	*p*-value	Eta Square
Accelerations (m/min)	7.85 ± 1.65^c^	6.35 ± 1.66^c^	7.25 ± 1.43^c^	14.3 ± 4.15	30.9	<.001	.704
Decelerations (m/min)	7.14 ± 1.25^a^^,^^b^^,^^c^	5.63 ± 0.77^c^	5.88 ± 1.18^b^^,^^c^	10.0 ± 2.46	26.2	<.001	.668
Sprints (m/min)	16.0 ± 2.29^a^^,^^b^	2.83 ± 0.93^c^	5.29 ± 1.21^b^^,^^c^	19.8 ± 4.15	143	<.001	.916

Mid-court exercises (20 × 20 m); three-quarters court exercises (¾ court) (28 × 20 m), and full-court exercises involving situations of superiorities and inferiorities (40 × 20 m).

^a^ Significantly different than ¾ court.

^b^ Significantly different than Mid-court.

^c^ Significantly different than full court; *p* ≤ 0.05.

Significant differences with small to large effects were verified for acceleration distances among categories (*F* = 30.9, *p* < .001, *η*2 = .704). Regarding decelerations (m/min^−1^), significant differences with small to large effects were verified between groups (*F* = 26.2, *p* < .001, *η*2 = .668).

Finally, significant differences in sprint performance were observed, with effect sizes ranging from small to large (*F* = 143, *p* < .001, *η*2 = .916).

Table 4 displays descriptive data of the coefficient of intra-player variability of performance variables and comparisons across match and exercise categories.

**Table 4:** Descriptive statistics (mean ± SD) and repeated measures ANOVA results for coefficient of variation (CV) of performance variables across match and exercise categories.

CV of the performance variables	Match	Mid-court	¾ court	Full court	F	*p*-value	Eta Square
Accelerations (m/min)	23.3 ± 4.20^a^^,^^b^^,^^c^	77.9 ± 24.1	55.8 ± 12.0^b^	57.9 ± 12.3	45.7	<.001	.649
Decelerations (m/min)	21.9 ± 4.36^a^^,^^b^^,^^c^	79.7 ± 24.5	58.3 ± 15.0^b^	68.5 ± 15.2	47.2	<.001	.784
Sprints (m/min)	22.3 ± 3.81^a^^,^^b^^,^^c^	127.0 ± 0.29^c^	81.9 ± 14.9^b^^,c^	63.5 ± 11.8	87.4	<.001	.871

Mid-court exercises (20 × 20 m); three-quarters court exercises (¾ court) (28 × 20 m), and full-court exercises (40 × 20 m).

^a^ Significantly different than ¾ court.

^b^ Significantly different than Mid-court.

^c^ Significantly different than full court; *p* ≤ 0.05.

Significant differences in the coefficient of variation for the analysed performance variables were found in ACC, with effect sizes ranging from small to large (*F* = 45.7, *p* < .001, *η*2 = .649). Similarly, significant differences in DEC variability were observed between groups (*F* = 42.2, *p* < .001, *η*2 = .784). Finally, significant differences with large effects were found in the coefficient of variation for sprints across all groups (*F* = 87.4, *p* < .001, *η*2 = .871).

**Figure 2:**
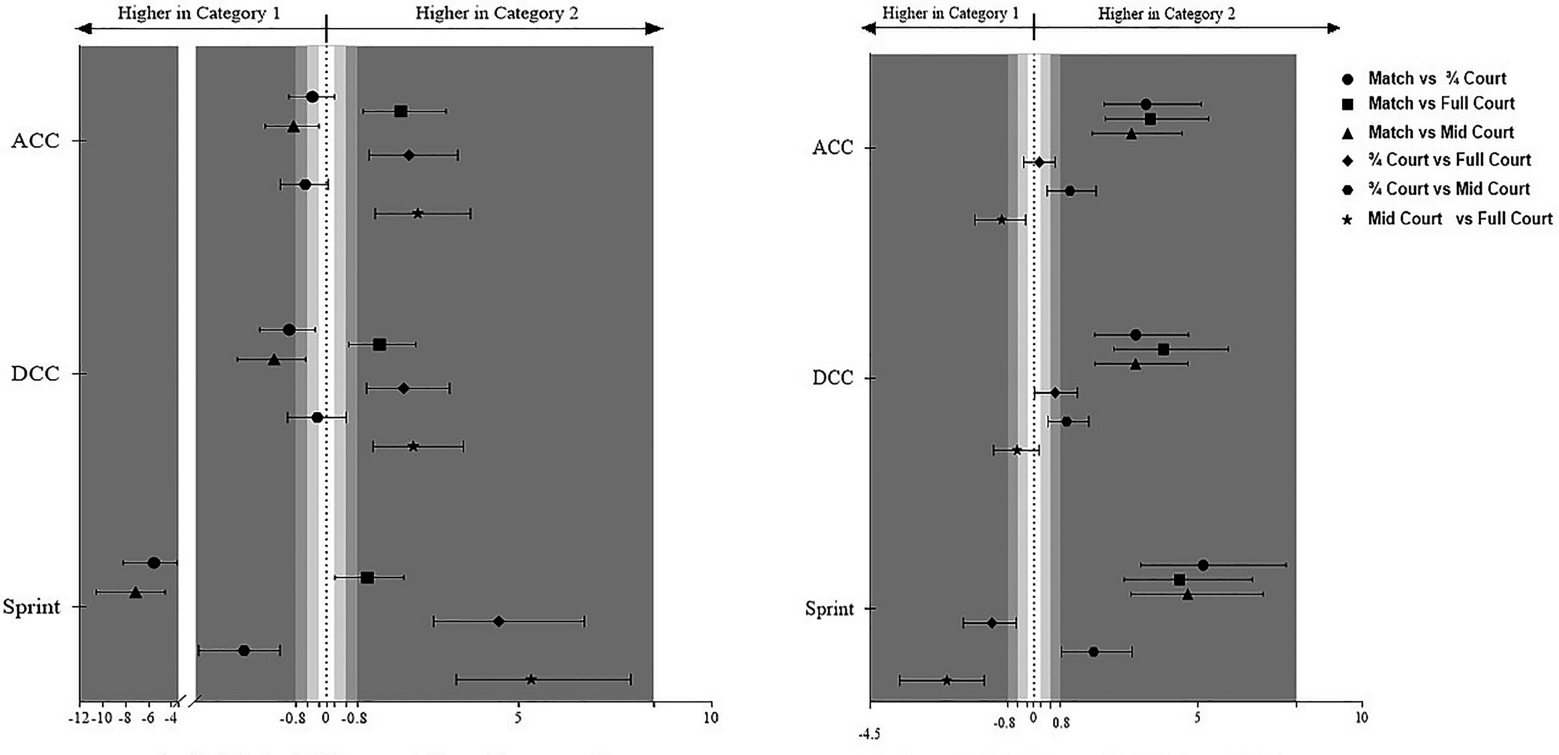
Cohen's *d*_unbiased_ differences. Left panel represent the performance differences between match and exercises categories. Right panel represent the intra players variability differences between match and exercises categories error bars represent 95% confidence intervals.

Figure 2 (right panel) represents the Cohen's d unbiased according to the intra-player variability match and exercise categories performances.

## Discussion

This study aims to compare high-intensity activities such as accelerations, decelerations, and high-speed running, across different training exercises and official match-play scenarios. As expected, full court training exercises presented the highest external load demands for acceleration, deceleration and sprinting. Notably, variability analysis revealed that match conditions exhibited the lowest fluctuations across all analyzed variables, suggesting a more stable and consistent physical demand during competitive play. These findings highlight the importance of considering both intensity and variability when designing training programs to optimize player performance.

Furthermore, these results align with previous research indicating that larger playing areas promote higher intensities (2) as the increased space facilitates individual movements and displacements (4, 9). For instance, players in possession have more space to engage in 1v1 situations, which in turn, due to the game's interdependent nature, compel defenders to adjust their positioning to cover the space (19). Similarly, while attacking players create passing lines or open spaces for teammates, defenders must continuously adapt to protect the available space. Defensive actions, such as marking, and offensive movements aimed at breaking defensive lines, often involve high-intensity accelerations, decelerations, and running (20).

However, it is important to acknowledge that playing space is not the only factor influencing physical demands. The number of players and specific exercise rules also play a crucial role in shaping players responses. Variations in formats, such as 2v2 vs. 3v3, the inclusion of jokers, or the presence of goals, can significantly alter movement patterns, tactical behaviours, and physical intensity. These interdependent interactions between opposing teams (21) likely contribute to the overall increase in external load, particularly in scenarios with larger playing areas. In this respect, full-court exercises provide more opportunities to explore disruptive actions during attacks while increasing defensive movements (22).

Interestingly, the full court presented higher values of external load when compared to the match. The use of different number of players and the possibility of exploiting numerical superiority in attack during some full-court training exercises (23) along with shooting actions occurring at a higher frequency, may explain the disparity in intensity between these training exercises and actual matches (11). As previously observed, the increase in space per player, combined with a reduction in the number of participants in the training exercises, could lead to a general increase in running distance and high-speed actions compared to the official match structure (4, 24).

While it might be expected that the three-quarters court format would show lower values for high-intensity ACC and DEC due to the smaller available playing area compared to other formats, the analysis revealed that mid-court exercises actually promoted the shortest distances at high-intensity ACC and DEC. The manipulation of relative area per player in each training task plays a key role in determining whether a task is more or less physically demanding, depending on the individual and collective tactical movements required (4). Although smaller formats generally result in reduced load (5), the physical stimulus is also influenced by other task constraints, such as the number of players involved. As shown in Table 1, while the three-quarters court format includes scenarios ranging from 2v2–4v4, the middle court format primarily involves 1v1–3v3 situations and incorporates the use of jokers. This combination of increased space per player and a lower numerical relationship likely contributed to the lowest high-intensity values observed in middle court exercises. In these scenarios, players may have more opportunities to destabilize their direct opponents and create goal-scoring situations due to the reduced distance to the target or numerical superiority (9, 23).

Meanwhile, the reduced sprint values in smaller court dimensions reflect the spatial constraints that limit the total distance covered in sprint actions. However, in futsal, the most frequent accelerations and decelerations occur over distances of less than 10 meters and at magnitudes below 3 m.s-2. Therefore, despite being less demanding in terms of high-intensity efforts per minute, mid-court training exercises should be used to develop short, fast movements with high frequency, as seen in the game (20).

However, beyond understanding the magnitude of effort for each training task, it is crucial to examine both intra- and inter-player variability within each type of training task and match (17). This can help better understand variations in players' load throughout the week and their preparation for competition (5, 25). In this context, dynamically understanding training loads and the variability accounted for in each training task's impact on individual physical performance is of paramount importance. It facilitates the design and planning of the most appropriate exercises, not only based on their level of effort but also on the level of variability in the efforts they promote. Consequently, this approach enables the development of performance-oriented training plans tailored to the specific needs of both the team and individual players (26).

In line with our expectations, the comparison between the training exercises and the match revealed distinct performance variability across exercise categories. Mid-court training exercises exhibited significantly higher variability compared to all other training formats and match scenarios. In contrast, match conditions demonstrated the lowest variability. These findings confirm that, even within the same training category, players can exhibit different levels of efforts across sessions or even between individuals. This variability suggests that effort levels may not always align with the expectations set by coaches. Such fluctuations highlight the frequent neglect of both intra-individual and inter-individual variability when analyzing training responses, particularly in more heterogeneous training tasks (16).

Results showed that match conditions exhibit the lowest variability in ACC, DEC, and sprint variables compared to training exercises. This result can be attributed to the higher stability in the rules and the numerical relation between players that participated in the training exercises and in the match. Matches are characterized by adherence to a shared tactical framework, where players operate within a structure dictated by the coach's strategy. Additionally, during matches, coaches can make continuous substitutions, which helps maintain a more consistent physical profile among players on the pitch (6). In contrast, training exercises are intentionally designed to target specific movement behaviors under controlled physical stimuli, often prioritizing certain tactical or technical objectives (23). Training exercises provide players with opportunities to explore different game environments, which naturally leads to broader variability in action and intensity metrics (16). Altogether, these factors may have contributed to the greater variation during training exercises resulting from differing intensities and objectives (27).

In opposition to the results of the magnitude of efforts, among training exercises, Middle court exercises exhibited the highest value of variability in ACC, DEC, and sprint performance, followed by three quarters and full court exercises. The confined space and the varied number of players involved could amplify the influence of individual decision-making and small-scale interactions, leading to variations on physical outputs (28). Conversely, full court exercises, despite promoting the highest overall intensities, showed relatively lower variability compared to middle and three quarters exercises. This may result from the structured nature of foul court drills designed to simulate match-specific scenarios and superiorities and inferiorities scenarios, leading to more predictable performance outputs, in line with match results.

Nevertheless, despite the inherent challenges of conducting research with elite teams and the data encompassing three competitive seasons, these findings should be interpreted with caution, as they are derived from a single club's context. Additionally, a limitation of this study is that exercise categories were defined solely based on pitch dimensions, despite incorporating different numbers of players and specific rules. These factors may have influenced the variability observed in external load responses. This study represents an initial exploratory approach to understanding the influence of space manipulation on the magnitude and variability of external load response. However, further research is required to compare the training exercises, taking into consideration not only the space of play but also the number of players involved, the numerical relations, or even the goals of the exercises.

## Practical implications

The findings suggest that full court exercises are particularly effective for developing high-intensity activities, including ACC, DEC, and sprint performance. Coaches aiming to replicate match demands should prioritize incorporating full court drills into training regimens. However, exercises in smaller court dimensions, such as middle and three quarters exercises, may be valuable for enhancing the frequency and variability of actions, players' adaptability, and decision-making under varying spatial constraints. The higher variability observed in these formats indicates their potential for fostering also physical aspects related to technical and tactical versatility. Finally, the low variability observed in match conditions emphasizes the importance of structured gameplay in achieving consistent performance levels. Training should balance high-intensity drills with variability-inducing exercises to optimize players' readiness for diverse match scenarios.

## Conclusion

This study underscores the critical interplay between spatial constraints, external load, and variability in futsal. Matches provide consistent intensity levels due to structured tactical frameworks and competitive dynamics, while training exercises, particularly in smaller court dimensions, exhibit greater variability. These findings highlight the importance of tailoring training programs to balance intensity, variability, and contextual relevance, ultimately optimizing athlete preparation for the multifaceted demands of competitive play. For the future, in order to better classify the training tasks and its effects, coaches should not only consider the playing area, but the number of players considered and its relative area. It allows a more precise classification of the effects of the training tasks.

## Data Availability

The raw data supporting the conclusions of this article will be made available by the authors, without undue reservation.
